# Synergistic Enhancement of Near-Infrared Emission in CsPbCl_3_ Host via Co-Doping with Yb^3+^ and Nd^3+^ for Perovskite Light Emitting Diodes

**DOI:** 10.3390/nano13192703

**Published:** 2023-10-04

**Authors:** Muhammad Amin Padhiar, Shaolin Zhang, Minqiang Wang, Noor Zamin Khan, Shoaib Iqbal, Yongqiang Ji, Nisar Muhammad, Sayed Ali Khan, Shusheng Pan

**Affiliations:** 1 School of Physics and Materials Science, Guangzhou University, Guangzhou 510006, China; amin.padhiar@gzhu.edu.cn (M.A.P.);; 2 Research Center for Advanced Information Materials (CAIM), Huangpu Research and Graduate School of Guangzhou University, Guangzhou 510555, China; 3 Key Lab of Si-based Information Materials & Devices and Integrated Circuits Design, Department of Education of Guangdong Province, Guangzhou 510006, China; 4 Electronic Materials Research Laboratory, Key Laboratory of the Ministry of Education International Center for Dielectric Research Xi’an Jiaotong University, Xi’an 710049, Chinayongqiangji@pku.edu.cn (Y.J.); 5 State Key Laboratory for Artificial Microstructure and Mesoscopic Physics, School of Physics, Peking University, Beijing 100871, China; 6 Hefei National Research Center for Physical Sciences at the Microscale and Department of Physics, University of Science and Technology of China, Hefei 230026, China; laiba@mail.ustc.edu.cn; 7 Department of Chemistry and Chemical Engineering, Rutgers, The State University of New Jersey, Piscataway, NJ 08854, USA

**Keywords:** perovskite nanocrystals, NIR Emission, co-doping, LEDs, external quantum efficiency

## Abstract

Perovskite nanocrystals (PeNCs) have emerged as a promising class of luminescent materials offering size and composition-tunable luminescence with high efficiency and color purity in the visible range. PeNCs doped with Yb^3+^ ions, known for their near-infrared (NIR) emission properties, have gained significant attention due to their potential applications. However, these materials still face challenges with weak NIR electroluminescence (EL) emission and low external quantum efficiency (EQE), primarily due to undesired resonance energy transfer (RET) occurring between the host and Yb^3+^ ions, which adversely affects their emission efficiency and device performance. Herein, we report the synergistic enhancement of NIR emission in a CsPbCl_3_ host through co-doping with Yb^3+^/Nd^3+^ ions for perovskite LEDs (PeLEDs). The co-doping of Yb^3+^/Nd^3+^ ions in a CsPbCl_3_ host resulted in enhanced NIR emission above 1000 nm, which is highly desirable for NIR optoelectronic applications. This cooperative energy transfer between Yb^3+^ and Nd^3+^ can enhance the overall efficiency of energy conversion. Furthermore, the PeLEDs incorporating the co-doped CsPbCl_3_/Yb^3+^/Nd^3+^ PeNCs as an emitting layer exhibited significantly enhanced NIR EL compared to the single doped PeLEDs. The optimized co-doped PeLEDs showed improved device performance, including increased EQE of 6.2% at 1035 nm wavelength and low turn-on voltage. Our findings highlight the potential of co-doping with Yb^3+^ and Nd^3+^ ions as a strategy for achieving synergistic enhancement of NIR emission in CsPbCl_3_ perovskite materials, which could pave the way for the development of highly efficient perovskite LEDs for NIR optoelectronic applications.

## 1. Introduction

The distinctive characteristics of metal halide perovskite nanocrystals (PeNCs), including their ability to tune bandgaps, exhibition of solution-phase photoluminescence (PL), and compatibility with diverse manufacturing techniques, make them highly promising for light-emitting applications. PeNC-based light-emitting diodes (PeLEDs) have been able to achieve remarkable external quantum efficiencies (EQEs) in emitting green, red, and blue light [1,2,3,4].

Regrettably, PeNCs are constrained by a bandgap range of approximately 2.9–1.7 eV (equivalent to 410–700 nm), which confines their capabilities to the visible spectrum [5,6,7,8,9]. This limitation results in their PL properties being restricted to this specific range. Consequently, PeNCs are not able to emit light beyond this range [10,11,12,13], which makes them unsuitable for applications that require operation in the near-infrared (NIR) region. This includes night-vision devices, optical communications, biomedical imaging, and surveillance. NIR PeLEDs are highly desirable for such applications [14,15,16,17,18,19]. Doping Yb^3+^ ions into CsPbCl_3_ PeNCs with emission at around 900 nm is an effective strategy for tuning the emission of PeNCs hosts to the NIR region [12,13,20]. However, achieving efficient NIR emission PeLEDs remains challenging [21]. More importantly, single-doped Yb^3+^ PeLEDs face significant challenges with weak NIR emission and low EQE. This is primarily due to undesired resonance energy transfer (RET) occurring between the sensitizers and Yb^3+^ ions, which adversely affects their emission efficiency and device performance. Yb^3+^ ions have only two energy levels of 2F_5/2_ and 2F_7/2_, compared to other trivalent rare-earth ions (such as Tm^3+^, Ho^3+^, Pr^3+^, Nd^3+^, and Er^3+^) which typically have more than two valence f-electronic states. Therefore, improving the NIR emission efficiency of Yb^3+^ in perovskites has become a hot issue [22,23,24,25,26]. The potential to enhance the NIR emission efficiency of Yb^3+^ doped CsPbCl_3_ PeNCs by employing various approaches, including ligand-assisted reprecipitation, doping concentration optimization, and co-doping with other rare-earth ions, has been studied. Such advancements could have significant implications for the development of highly efficient NIR-emitting perovskite-based optoelectronic devices [27]. Recently, scientists have discovered that the addition of co-doped Bi^3+^ or Na^+^ ions can significantly enhance the NIR emission associated with Yb^3+^. A recent study conducted by Nag et al. revealed that co-doping of Bi^3+^ ions in PeNCs has the ability to increase the excitation efficiency and allow them to serve as sensitizers for NIR emission of Yb^3+^ ions. As a result, the NIR emission of Yb^3+^ was substantially improved [28,29]. Liu et al. reported near-infrared afterglow and related photochromism from solution-grown PeNCs [30]. Additionally, Zhang et al. discovered that introducing Na^+^ ions can also increase the NIR luminescence of Yb^3+^ ions. By inducing a breakdown in the local site symmetry of the PeNCs, the absorption significantly increases due to Na^+^, which leads to efficient energy transfer, ultimately populating the 2F_5/2_ state of Yb^3+^. Similarly, Wu et al. demonstrated that the Bi^3+^/Yb^3+^ co-doped PeNCs matrix enhances the NIR emission of Yb^3+^ ions through energy transfer from self-trapped excitons (STEs) [31,32]. Recently, the quantum cutting effect of Yb^3+^ has also been reported. However, the optimization of NIR-range devices involves post-passivation of PeNCs’ surface by using benzyl thiocyanate (BTC) [33]. Despite the success in achieving NIR EL from CsPbCl_3_ PeNCs through single doping of Yb^3+^, the potential for achieving NIR EL through the co-doping of trivalent lanthanide ions (Ln^3+^) in host CsPbCl_3_ PeNCs has not been realized, and this area of research remains largely unexplored. [34,35,36]. The primary challenge in achieving highly efficient NIR emission of single-doped Yb^3+^ in PeNCs is the low sensitization efficiency, which is caused by undesirable RET between the sensitizer and Yb^3+^ ions. This issue requires an urgent resolution. According to Förster–Dexter energy transfer theories, achieving a high energy transfer efficiency relies on the degree of overlap between the sensitizer emission spectrum and the activator Yb^3+^ absorption profile. As such, regulating the emission spectrum of the sensitization center to achieve better resonance with Yb^3+^ is worth exploring [37]. Herein, we achieved a high-efficiency NIR emission by using Yb^3+^/Nd^3+^ co-doping in CsPbCl_3_ host PeNCs. By virtue of co-doping Yb^3+/^Nd^3+^, can address the issue of weak NIR emission and undesired RET between the host and Yb^3+^ ions, which limit their emission efficiency and device performance. The Nd^3+^ ions act as energy donors, and when co-doped with Yb^3+^ ions, Nd^3+^ ions can transfer excitation energy to the Yb^3+^ ions. This process enhances the population of Yb^3+^ excited states and thus improves their NIR emission efficiency. The PeLEDs incorporating the co-doped CsPbCl_3_ PeNCs emissive layer exhibited a maximum external quantum efficiency of 6.2%, which is approximately 2.8 times higher than that of devices based on undoped CsPbCl_3_ PeNCs. These findings demonstrate the significant potential of Yb^3+^ and Nd^3+^ co-doping in enhancing the NIR emission and device performance of PeLEDs.

## 2. Experimental Section

### 2.1. Chemical and Materials

Caesium acetate (CsOAc) (99.99%), Lead acetate trihydrate [Pb(OAc)_2_·3H_2_O] (99.99%), Ytterbium acetate trihydrate [Yb(OAc) 3H_2_O] (99.9%), and Neodymium (III) bromide hexahydrate (NdBr_3_·6H_2_O) (99.9%) were purchased from Sigma Aldrich. Chlorotrimethylsilane (TMS-Cl) (≥99%) was obtained from Aladdin chemicals. Oleic acid (OA) (90%), Oleylamine (OLA) (70%), and 1-Octadecene (ODE) (90%) were purchased from Sigma-Aldrich. Hexane (99.9%), Ethyl acetate (99%), and Ethanol (99%) were obtained from Aladdin chemicals. PEDOT:PSS solution, poly-TPD, PVK, and TPBi were purchased from Xi’an Polymer Light Technology Corp. All the chemicals were used without further purification.

### 2.2. Synthesis of Single Doped and Co-Doped PeNCs

The CsPbCl_3_/Yb^3+^-doped PeNCs were synthesized using a modified hot-injection method described in a previous report [38]. In a 25 mL three-neck flask, CsOAc (53.7 mg, 0.28 mmol), Pb(OAc)_2_3H_2_O (75.9 mg, 0.20 mmol), and Yb(OAc)_2_3H_2_O (4.9 mg, 0.02 mmol) were mixed with OA (0.5 mL), OAm (1.0 mL), and ODE (5.0 mL). The synthesis of co-doped CsPbCl_3_/Yb^3+^/Nd^3+^ PeNCs involved varying the Nd-doping concentrations by adjusting the amount of NdBr_3_·6H_2_O (III) precursor used. The feeding amount of Yb(OAc)_2_·3H_2_O remained constant at 0.02 mmol, while the feeding amounts of NdBr_3_·6H_2_O (III) for the different samples were set at 0, 0.02, 0.04, 0.06, and 0.08 mmol, respectively. The solution was degassed at room temperature for 10 min and then heated to 120 °C for 1 h. During this heating process, all precursors completely dissolved, resulting in a transparent mixture. The reaction vessel was then refilled with N_2_ and further heated to 200 °C. Once the solution reached this temperature, a swift injection of 0.20 mL TMS-Cl caused the solution to immediately become turbid. After 10 s, the solution was rapidly cooled to room temperature using an ice bath.

### 2.3. PeLED Device Fabrication

The fabrication of the PeLED utilized a modified method originally reported by Chiba et al. [39]. The PeLED structures used in the experiment were as follows: a layer of indium tin oxide (ITO) with a thickness of 130 nm, followed by a layer of poly(3,4-ethylenedioxythiophene):polystyrene sulfonate (PEDOT:PSS) with a thickness of 40 nm. On top of that, there was a perovskite layer of CsPbCl_3_:Yb^3+^ (or CsPbCl_3_:Yb^3+^/Nd^3+^) with a thickness of 70 nm. Next, there was a layer of 1,3,5-tri(m-pyridyl-phenyl)benzene (TmPyPB) with a thickness of 30 nm, followed by a 1 nm-thick layer of LiF and a 100 nm-thick layer of aluminum (Al). To prepare the ITO-coated glass substrates, they were cleaned using a spin rinsing system and treated with deionized water and UV-ozone for 10 min. A solution of PEDOT:PSS with Nafion (55 wt %) was spin-coated onto the cleaned ITO-coated glass substrate and then annealed at 150 °C for 10 min, resulting in a 40 nm-thick layer of PEDOT:PSS. In a N_2_-filled glovebox, a layer of 1,3,5-tris(N-phenylbenzimidazol-2-yl)benzene (TPBi) with a thickness of 30 nm, a 1 nm-thick layer of lithium quinolate (Liq), and a 100 nm-thick layer of aluminum (Al) were deposited onto the PEDOT:PSS/Nafion layer using thermal evaporation under high vacuum conditions (∼10−5 Pa). Finally, the fabricated PeLEDs with an active area of 2 mm^2^ were encapsulated using epoxy glue and glass covers in the N_2_-filled glovebox.

### 2.4. Characterization

The morphology of the PeNCs synthesized in this study was investigated using high-resolution transmission electron microscopy (TEM) with a JEOL JEM-F200 instrument. The chemical composition of the samples was analyzed using an Energy Dispersive Spectrometer (EDS) detector (Oxford X-MAX 65) connected to a transmission electron microscope. The size distribution histograms were obtained by analyzing the samples with a (Zetasizer Nano) (ZSE) instrument. X-ray diffraction (XRD) patterns were recorded using a (D8-Advance) instrument with Cu Kα (λ = 1.5406 Å) radiation, covering the 2θ range of 10–60°. The X-ray photoelectron spectroscopy (XPS) spectrum was analyzed using a (Thermo Fisher ESCALAB-670B Xi^+^) instrument. The ultraviolet-visible (UV-vis) absorption spectra were measured using a (Perkin Elmer Lambda 950) spectrophotometer. Ultraviolet photoelectron spectroscopy (UPS) measurements were carried out with a (Thermo Fisher) Scientific Theta probe, utilizing a He I UV source (21.22 eV) under a high vacuum of approximately 10^−6^ Pa and maintaining an operating voltage of −6 V. The photoluminescence (PL) spectra and time-resolved PL (TRPL) decay curves were collected using an (Edinburgh Instruments FLS-980) spectrometer equipped with a 450 W xenon lamp. The visible and near-infrared (NIR) luminescence signals were detected using a photomultiplier (PMT) (Hamamatsu, R928P) and a liquid nitrogen-cooled PMT (Hamamatsu, R5509-72), respectively. The absolute photoluminescence quantum yields (PLQYs) were measured using an integrating sphere (Edinburgh Instruments) connected to the FLS-980 spectrometer. The current density-voltage (J-V) characteristics of the PeLEDs were determined using a Keithley 2400 source meter, while the front-face electroluminescence (EL) radiance, EL spectra, and EQE were measured with a Photoresearch Spectra Scan spectrometer (PR745) simultaneously.

## 3. Results and Discussion

**Figure 1:**
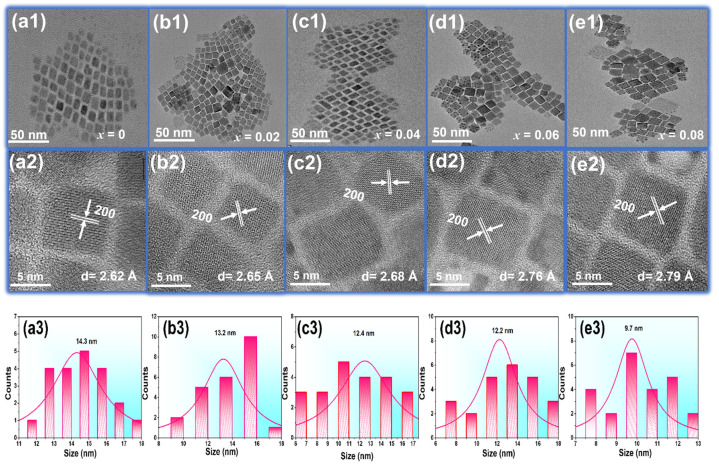
(**a1**–**e1**) TEM photographs of the CsPbCl_3_/Yb^3+^/*_x_*Nd^3+^ PeNCs (*x* = 0, 0.02, 0.04, 0.06, 0.08). (**a2**–**e2**) the corresponding HRTEM images. (**a3**–**e3**) Size distribution histograms of the corresponding PeNC samples.

Transmission electron microscopy (TEM) measurements were performed to investigate the morphology and structure of CsPbCl_3_ PeNCs doped with Yb^3+^ and Nd^3+^ ions. The samples were synthesized with a fixed concentration of Yb^3+^ ions and varying concentrations of Nd^3+^ ions ranging from (*x* = 0, 0.02, 0.04, 0.06, 0.08) TEM images are shown in Figure 1(a1–e1) CsPbCl_3_/Yb^3+^/*_x_*Nd^3+^ revealed a structure similar to traditional CsPbCl_3_ PeNCs [39]. As the concentration of Nd^3+^ ions increased in the CsPbCl_3_/Yb^3+^/Nd^3+^ PeNCs, a slight reduction in the lattice size was observed which is consistent with XRD data. This can be attributed to the substitution of Yb^3+^/Nd^3+^ ions for Pb^2+^ ions in the CsPbCl_3_ lattice. Since Pb^2+^ ions have a larger ionic radius (1.19 Å) compared to Yb^3+^ ions (0.90 Å) and Nd^3+^ ions (1.02 Å), when Yb^3+^ and Nd^3+^ ions replace Pb^2+^ ions in the lattice, the resulting lattice size contracts slightly. This is because the smaller Yb^3+^ and Nd^3+^ ions occupy the lattice positions that were previously occupied by larger Pb^2+^ ions. The effect of Nd^3+^ concentration on the lattice size is likely influenced by a combination of factors. These factors include the increased likelihood of Nd^3+^ ions substituting for Pb^2+^ ions and the increased interactions between Nd^3+^ ions and the surrounding lattice as the concentration of Nd^3+^ ions increase. The interplanar distances of the crystalline structure’s (200) planes, as per cubic reference PDF #180366 [39], are illustrated in Figure 1(a2,a3). It is worth noting that the fixed concentration of Yb^3+^ ions may also contribute to the lattice contraction. The substitution of Yb^3+^ ions for Pb^2+^ ions could also potentially lead to lattice contraction. Moreover, the presence of Yb^3+^ ions can also influence the interaction between Nd^3+^ ions and the lattice, thereby contributing to the overall lattice contraction [40]. When the Nd^3+^ concentration reaches *x* = 0.08, the cubic morphology of the CsPbCl_3_ PeNCs begins to deform. This deformation indicates that additional doping beyond this concentration will have a substantial impact on the crystalline structure of the PeNCs. The histograms of the prepared PeNCs revealed an average particle size of approximately 14.3 nm to 9.7 nm with varying concentrations as shown in Figure 1(a3–e3). These TEM measurements provide insights into the morphology and structure of CsPbCl_3_ PeNCs doped with Yb^3+^ and Nd^3+^ ions, which can aid in the development of PeLEDs with synergistic enhancement of near-infrared emission.

**Figure 2:**
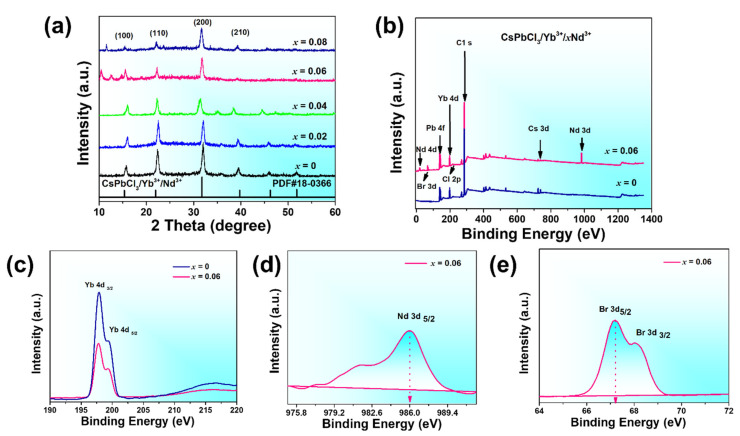
(**a**) XRD patterns of CsPbCl_3_/Yb^3+^/*_x_*Nd^3+^ PeNCs (*x* = 0, 0.02, 0.04, 0.06, 0.08). (**b**) XPS Spectra of CsPbCl_3_/Yb^3+^/*_x_*Nd^3+^ PeNCs (*x* = 0, 0.06,). (**c**–**e**) High resolution XPS spectra of Yb 4d, Nd 3d, and Br 3d, respectively.

X-ray diffraction (XRD) measurements were further conducted to examine and validate the previously performed TEM results. The XRD patterns of CsPbCl_3_/Yb^3+^/*_x_*Nd^3+^ (*x* = 0, 0.02, 0.04, 0.06, 0.08) were analyzed, revealing distinct diffraction peaks at specific 2θ angles. These angles were approximately 15.8°, 22.3°, 32.0°, and 40.0°, corresponding to the crystal planes (100), (101), and (200) of the cubic Pm3m CsPbCl_3_ space group and PDF#(18-0366), respectively [39]. Figure 2a displays the XRD peaks of the respective samples. As the concentration of Nd^3+^ ions increased, the diffraction peaks shifted slightly to higher angles. This trend is consistent with the TEM measurements, which showed a slight change in lattice size as Nd^3+^ concentration increased [40]. When the Nd^3+^ concentration reaches (*x* = 0.08), the diffraction peaks exhibit weakened intensity, indicating a significant decline in crystal quality and a transition to a non-cubic phase at higher levels of Nd^3+^ doping. This observation suggests that nominal Nd^3+^ doping preserves the cubic structure in comparison to higher levels of Nd^3+^ doping. This is consistent with the TEM measurements, which showed a deformation of cubic morphology at this concentration. Table 1 provides the detailed positions of XRD peaks corresponding to different concentrations.

X-ray photoelectron spectroscopy (XPS) measurements were performed to investigate the surface chemistry of CsPbCl_3_ PeNCs doped withYb^3+/^*_x_*Nd^3+^ ions (where *x* = 0 and 0.06), as illustrated in Figure 2b. The XPS spectra of the co-doped sample exhibited distinct peaks corresponding to the core levels of Cs 3d, Pb 4f, Cl 2p, Br 3d, Yb 4d, and Nd 3d. This finding aligns with previous reports and provides compelling evidence for enhanced Pb-Yb-Nd interactions in the co-doped system [37]. Specifically, the Yb 4d peaks were located at an approximate energy of 197 eV, confirming the successful incorporation of Yb^3+^ ions at the surface level, as demonstrated in Figure 2c. Moreover, additional peaks corresponding to Nd 3d and Br 3d were observed at energies of 986 eV and 67.2 eV, respectively, exclusively in the co-doped sample [41]. These peaks were absent in the spectra of the single doped samples, as illustrated in Figure 2d,e. Consequently, these findings strongly support the presence of Nd^3+^ ions in PeNCs, directly correlated with the doping concentration, particularly in the *x* = 0.06 sample. Additional evidence of the surface chemistry of the CsPbCl_3_ PeNCs can be found in Figure S1a–c, which presents the XPS peaks corresponding to the core levels of Cs 3d, Pb 4f, and Cl 2p.

**Figure 3:**
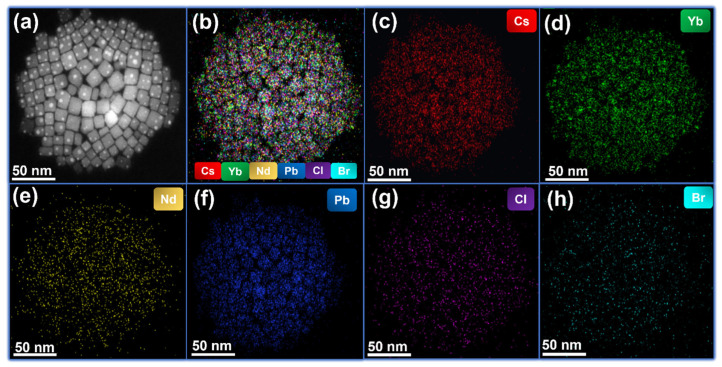
(**a**) HAADF-STEM image of CsPbCl_3_/Yb^3+^/*_x_*Nd^3+^ PeNCs. (**b**–**h**) Elemental mappings of Cs, Yb, Nd, Pb, Cl, and Br elements in the CsPbCl_3_/Yb^3+^/*_x_*Nd^3+^ PeNCs.

The synergistic co-doping effect of Yb^3+^ and Nd^3+^ on CsPbCl_3_ PeNCs was further investigated using high-angle annular dark-field scanning transmission electron microscopy (HAADF-STEM) imaging, elemental mapping, and energy-dispersive X-ray spectroscopy (EDS). Figure 3a displays the HAADF-STEM image of the co-doped PeNCs with an Nd^3+^ concentration of (*x* = 0.06), revealing a cubic crystal structure, which is consistent with the TEM photographs shown in Figure 1. To investigate the distribution of each element within the PeNCs, elemental mapping of Cs, Pb, Cl, Br, Yb, and Nd was performed. Cs, Pb, Br and Cl were found to be uniformly distributed throughout the PeNCs, as indicated by their consistent presence in the elemental maps. In contrast, Yb and Nd were predominantly localized in the center of the nanocrystals, as depicted in Figure 3b–h. The elemental mapping results confirm that the co-doped PeNCs contain all the intended elements, as the maps display the presence of Cs (red), Yb (green), Nd (yellow), Pb (blue), Cl (purple), and Br (cayenne) within the sample. Furthermore, EDS analysis was conducted to determine the atomic percentages of Cs, Pb, Cl, Br, Yb, and Nd of the corresponding samples. The EDS signals and corresponding atomic percentages are presented in Figure S2. The analysis revealed that the atomic percentages of Cs, Pb, Cl, Br, Yb, and Nd were 17%, 12%, 19.7%, 11%, 7.8%, and 4.5%, respectively. Based on the findings from the HAADF-STEM imaging, elemental mapping, and EDS measurements, it can be concluded that the co-doping of Yb^3+^ and Nd^3+^ ions into the CsPbCl_3_ PeNCs was successful.

**Figure 4:**
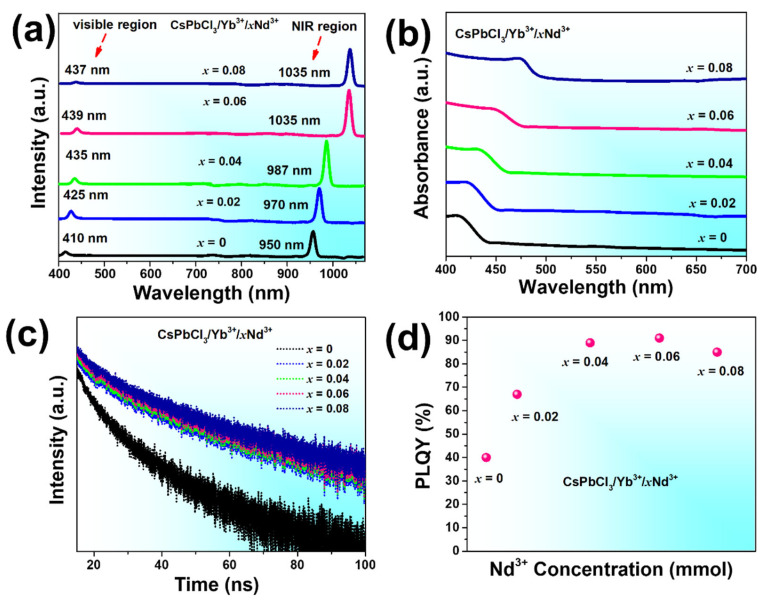
Optical characterization of CsPbCl_3_/Yb^3+^/*_x_*Nd^3+^ PeNCs (*x* = 0, 0.02, 0.04, 0.06, 0.08). (**a**) PL spectra (**b**) absorbance spectra. (**c**) TRPL curves. (**d**) absolute PLQY of the corresponding samples in the visible region.

Next, we studied the optical characteristics and synergistic enhancement of near-infrared emission in the CsPbCl_3_ host through co-doping with Yb^3+^ and Nd^3+^ PeNCs. The emission spectra of PeNCs were examined at a wavelength of 370 nm, encompassing the visible and NIR regions. In the PL analysis of CsPbCl_3_/Yb^3+^/*_x_*Nd^3+^(*x* = 0, 0.02, 0.04, 0.06, 0.08), the emission spectrum exhibits a prominent peak centered around 410 nm, which corresponds to the band-edge host emission of CsPbCl_3_, as depicted in Figure 4a. Additionally, a distinct peak is observed at approximately 950 nm, representing the NIR emission of Yb^3+^. Conversely, in the co-doped CsPbCl_3_ system with Yb^3+^/*_x_*Nd^3+^ (*x* = 0.02, 0.04, 0.06, and 0.08), the PL emission spectrum displays a host emission peak ranging from 425 nm to 437 nm, as illustrated in Figure 4a. Moreover, the PL emission spectra show a significant enhancement in the NIR emission from 970 nm to 1035 nm as the concentration of Nd^3+^ increases as shown in Figure 4a. The enhancement is due to the increased population of excited Nd^3+^ ions and improved energy transfer (ET) efficiency from CsPbCl_3_ to Yb^3+^ facilitated by Nd^3+^ ions, reducing undesired RET between the sensitizers and Yb^3+^ ions leading to a redshift by 85 nm. The intensity of the single doped NIR peak is relatively low compared to the co-doped NIR peak as shown in Figure S3, indicating that the energy transfer process from CsPbCl_3_ to Yb^3+^ is relatively inefficient in single-doped sample. The enhancement of the NIR emission is also accompanied by a decrease in the intensity of the band-edge emission of CsPbCl_3_, indicating that the ET process from CsPbCl_3_ to Yb^3+^ is more efficient in co-doped samples. Moreover, the PL analysis shows that as the concentration of Nd^3+^ ions increase, the intensity of the NIR emission peak increases until a certain point, after which it starts to decrease. This behavior is attributed to the competing effect of the ET from Nd^3+^ ions to Yb^3+^ ions and the formation of nonradiative centers at high concentrations of Nd^3+^ ions [42,43]. Therefore, there is an optimal concentration of Nd^3+^ ions for achieving the highest NIR emission intensity in co-doped CsPbCl_3_ PeNCs. Furthermore, in order to thoroughly comprehend the ET mechanism from to Yb^3+^ ions in Nd^3+^ systems, we conducted an analysis of the absorption spectra of CsPbCl_3_/Yb^3+^/*_x_*Nd^3+^ (*x* = 0, 0.02, 0.04, 0.06, 0.08) PeNCs samples. Moreover, we observed a distinct shoulder shift in the absorption spectra of the PeNCs, as illustrated in Figure 4b, which exhibited a correlation with the observed pattern in the PL spectra. This significant shift can likely be attributed to the intricate interactions occurring between the Nd^3+^ ions and Yb^3+^ ions within the material. These interactions facilitate efficient ET from the Yb^3+^ ions, contributing to the overall ET process. These results suggest that the incorporation of Nd^3+^ doping has a slight impact on the bandgap of the PeNCs. To validate this finding, we utilized a Tauc plot to calculate the bandgaps of each sample [44]. The observed shoulder shift in the absorption spectra suggests a modification of the absorption edge and absorption onset, indicative of the bandgap modification. We have observed a slight decrease in the bandgap: the calculated bandgaps of the corresponding samples are 3.14 eV, 3.2 eV, 3.1 eV, 3.01 eV, and 3.1 eV, respectively. This further supports the influence of the Nd^3+^ dopant on the electronic structure of the material as shown in Figure S4a–e. In order to investigate the influence of Nd doping on the ET kinetics of PeNCs, fluorescence decay measurements were performed. The time-resolved photoluminescence (TRPL) spectra of the samples were obtained at different Nd^3+^ feeding ratios, as shown in Figure 4c. To determine the lifetimes of each sample, a bi-exponential decay equation was employed.

(1) $$I=B_{1}\exp\left(-\frac{t}{\tau_{1}}\right)+B_{2}\exp\left(-\frac{t}{\tau_{2}}\right)$$

(2) $$\tau_{avu}=\frac{B_{1}\tau_{1}^{2}+B_{2}\tau_{2}^{2}}{B_{1}\tau_{1}+B_{2}\tau_{2}}$$

The lifetime ($B_{1}$*)* represents exciton radiative recombination, while the extended lifetime ($B_{2}$) is associated with surface radiative defect recombination [35]. According to Table S1, it is observed that with an increase in the Nd^3+^ ratio from 0 to 0.08, the proportion of ($B_{1}$) increases from 70.53% to 96.59%, while the contribution of ($B_{2}$) decreases from 30.45% to 11.82%. This indicates the suppression of surface traps [45,46]. The efficiency of energy transfer (*η_ET_*) to Yb^3+^ ions can be determined using Equation (3).

(3) $$\eta_{ET}=\left(1-\frac{\tau }{\tau_{0}}\right)$$

This equation utilizes the lifetimes’ (*τ* and *τ_0_*) emission in the presence and absence of Nd^3+^, respectively. It is evident that the efficiency of ET gradually increases with the rising Nd^3+^ content, reaching above 60%. The effective ET for each sample is depicted in Figure S5. The remarkable ET can be attributed to two primary factors. Firstly, both Yb^3+^ and Nd^3+^ ions occupy space within the CsPbCl_3_ host, resulting in a short distance between the Yb^3+^ sensitizer and Nd^3+^ activator. This proximity enhances the probability of ET occurring [37]. The excited Nd^3+^ states subsequently undergo radiative recombination processes, leading to the emission of photons and the enhancement of PLQY. As a result, PLQY in the visible region of the corresponding samples shows a significant increase from 40% to 90.5%, as illustrated in Figure 4d.

**Figure 5:**
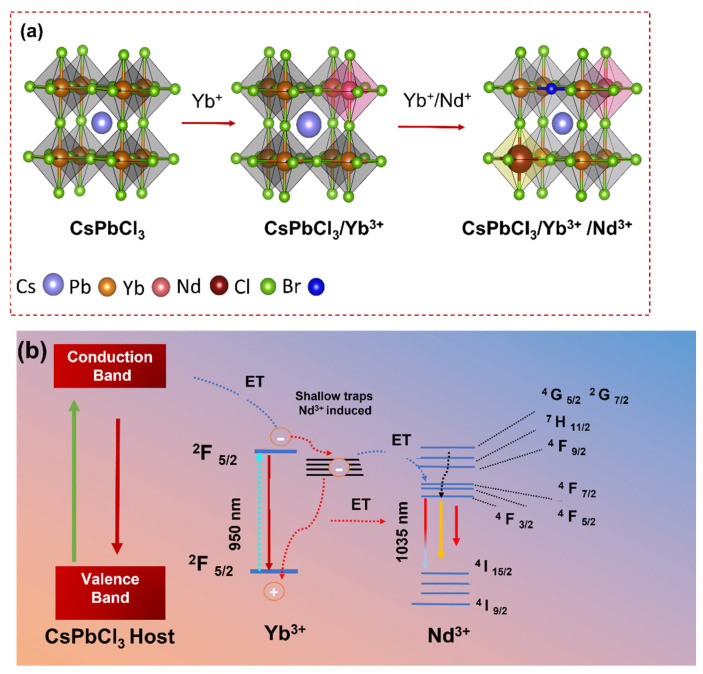
(**a**) Schematic illustration of Yb^3+^ and Nd^3+^ substituting Pb^2+^ in the CsPbCl_3_ lattice. (**b**) Schematic diagram of energy levels and energy transfer processes in Yb^3+^/Nd^3+^ co-doped CsPbCl_3_.

Figure 5a depicts the schematic representation Yb^3+^ and Nd^3+^ substituting Pb^2+^ in the CsPbCl_3_ host PeNCs. The chemical expressions of single and co-doped PeNCs are provided in Equations (4) and (5), respectively.

2CsOAc + Pb(OAc)_2_3H_2_O + Yb(OAc)_2_3H_2_O→CsPbCl_3_/Yb^3+^ (4)

2CsOAc + Pb(OAc)_2_3H_2_O + Yb(OAc)_2_3H_2_O + NdBr_3_6H_2_O→CsPb(Br,Cl)_3_/Yb^3+/^Nd^3+^ (5)

The ET efficiency of the CsPbCl_3_/Yb^3+^/Nd^3+^ perovskite system was further explained using an ET diagram, as depicted in Figure 5b. The ET diagram provides a graphical representation of the ET processes occurring within the system based on the experimental results. It was found that the NIR emission of Yb^3+^ ions are significantly enhanced by the presence of Nd^3+^ ions. The ET process from Nd^3+^ to Yb^3+^ is attributed to the efficient cross-relaxation process between the ^4^F_3/2_ level of Nd^3+^ and the ^2^F_5/2_ level of Yb^3+^. This process results in the population of the ^2^F _5/2_ level of Yb^3+^, which then emits NIR radiation at 1035 nm. The enhancement of the NIR emission of Yb^3+^ ions in the CsPbCl_3_:Yb^3+^/Nd^3+^ perovskite was also observed in the external EQE measurements. The EQE of the co-doped perovskite was found to be higher than that of the CsPbCl_3_/Yb^3+^ single-doped perovskite. This result indicates that the presence of Nd^3+^ ions improve the ET efficiency, resulting in an increased NIR emission [37]. The cross-relaxation process between Nd^3+^ and Yb^3+^ ions is an efficient ET mechanism in this system, which can be used to design and develop efficient NIR-emitting perovskite materials for optoelectronic applications [27].

**Figure 6:**
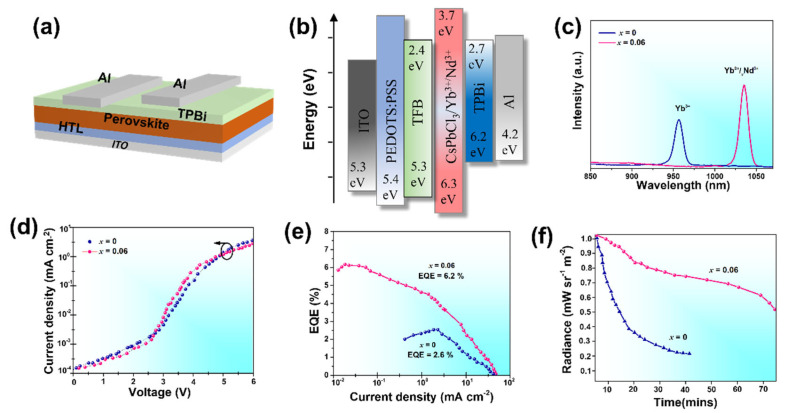
(**a**) Structure of the PeLED device, (**b**) Energy diagram illustration, (**c**) EL spectra, (**d**) Characteristics of current density, luminance, and voltage, and (**e**) Efficiency-Current relationship (EQE)−current density characteristics of PeLEDs. (**f**) Operational lifetime of PeLEDs.

To investigate the impact of doping on PeLED performance, CsPbCl_3_/Yb^3+^/*_x_*Nd^3+^ (*x* = 0, 0.06) PeNCs were employed as the light-emitting layer for LED fabrication. Figure 6a,b illustrate the structure and energy diagram of the PeLED. The valence bands of (*x* = 0, 0.06) were determined through ultraviolet photoelectron spectroscopy (UPS) conducted in an open atmosphere (Figure S6a,b). It was observed that the energy position of (*x* = 0.06) slightly decreased due to the incorporation of the Nd^3+^ dopant. The CsPbCl_3_/Yb^3+^/*x*Nd^3+^ (*x* = 0, 0.06) PeLEDs displayed EL wavelengths of approximately 950 nm and 1035 nm respectively, as indicated in Figure 6c. These emission wavelengths fall within the near-infrared range. The current density-luminance-voltage characteristics of the LEDs incorporating CsPbCl_3_/Yb^3+^/*_x_*Nd^3+^ (*x* = 0, 0.06) dopants are presented in Figure 6d. Remarkably, the LED with the Yb^3+^/*_x_*Nd^3+^ (0.06) dopant exhibited a lower turn-on voltage of only 3.0 V compared to the single Yb^3+^ dopant, which had a turn-on voltage of 3.5 V. This reduction in voltage can be attributed to a decrease in the hole injection barrier between TFB and Yb^3+^/*_x_*Nd^3+^-doped CsPbCl_3_ PeNCs. Figure 6e displays the measured EQE of the PeLEDs. It can be observed that the PeLEDs co-doped with Yb^3+^/*_x_*Nd^3+^ (*x* = 0.06) exhibited higher EQE compared to those doped with Yb^3+^. The PeLEDs co-doped with Yb^3+^/Nd^3+^ achieved a maximum EQE of 6.2%, which is approximately 2.38 times higher than the EQE of 2.6% obtained from the PeLEDs doped with Yb^3+^. The EQE reported here represents the highest value documented to date for wavelengths above 1000 nm. For comparison, Table 2 provides a compilation of EQE values for NIR OLEDs and PeLEDs reported in recent years. In addition, the PeLEDs co-doped with Yb^3+^/Nd^3+^ demonstrated higher luminance intensity compared to the PeLEDs doped with Yb^3+^, as shown in Figure S6c. The PeLED with co-doping achieved a maximum luminance of 140 cd m^−2^, which was significantly higher than that of the Yb^3+^ dopant. Furthermore, the operational lifetime of the NIR LEDs is presented in Figure 6f. The PeLED with Yb^3+^/Nd^3+^ dopant exhibited a longer lifetime compared to the PeLED based on the Yb^3+^ dopant due to a low turn-on voltage. This low turn-on voltage not only enhances the energy efficiency of the device but also mitigates the potential for degradation over time, ultimately leading to a substantial extension of its operational lifetime. These improvements in device efficiency can be attributed to the synergistic effects of Yb^3+^ and Nd^3+^ ions, which enhance ET efficiency and the population of excited Nd^3+^ ions. The enhanced EQE and NIR emission characteristics make these co-doped PeNCs promising candidates for applications such as photovoltaics and bioimaging, where efficient NIR emission is desired.

## 4. Conclusions

In conclusion, the co-doping of Yb^3+^ and Nd^3+^ ions in CsPbCl_3_ PeNCs has been demonstrated as an effective strategy to achieve synergistic enhancement of NIR emission. By incorporating Yb^3+^ and Nd^3+^ ions into the CsPbCl_3_ host, the NIR emission above 1000 nm was significantly enhanced, making it highly desirable for NIR optoelectronic applications. The presence of Nd^3+^ ions modified the emission spectrum, resulting in an 85 nm redshift. Furthermore, perovskite PeLEDs utilizing the Yb^3+^/Nd^3+^ co-doped CsPbCl_3_ PeNCs as the emitting layer exhibited substantially improved NIR EL compared to single-doped PeLEDs. The optimized co-doped PeLEDs demonstrated enhanced device performance, including increased EQE of 6.2% at a wavelength of 1035 nm. These findings highlight the potential of co-doping with Yb^3+^ and Nd^3+^ ions as a promising strategy for developing highly efficient PeLEDs for NIR optoelectronic applications.

## Figures and Tables

**Table 1:** The crystalline lattice constants of CsPbCl_3_/Yb^3+^/*x*Nd^3+^ PeNCs, Nd^3+^ ion doping concentrations (*x* = 0, 0.02, 0.04, 0.06, and 0.08), respectively.

Crystal Plane	Spacing Distance/Å PDF#18-0366	Spacing Distance/Å *x* = 0	Spacing Distance/Å *x* = 0.2	Spacing Distance/Å *x* = 0.4	Spacing Distance/Å *x* = 0.06	Spacing Distance/Å *x* = 0.08
100	5.6000	5.484	5.518	5.53	5.510	5.601
101	3.9600	3.80	3.846	3.875	3.945	3.967
200	2.7940	2.621	2.656	2.681	2.765	2.796

**Table 2:** Comparison of EQE values for as-investigated PeLED devices with NIR OLEDs and PeLEDs reported in recent years.

Device Type	Wavelength (nm)	EQE (%)	Reference
OLED	930	2.14%	[47]
OLED	840	3.8%	[48]
OLED	830	3.1%	[49]
PeLED	917	5.0%	[50]
PeLED	945	0.72	[51]
PeLED	950	3.8%	[52]
PeLED	1000	5.9%	[53]
PeLED	940	5.4%	[27]
PeLED	990	7.7%	[33]
PeLED	950	2.6%	This work
PeLED	1035	6.2%	This work

## Data Availability

All data that support the findings of this study are included within the article (and any Supplementary Files).

## Supplementary Materials

The following supporting information can be downloaded at: https://www.mdpi.com/article/10.3390/nano13192703/s1, Figure S1: High resolution XPS graph, Figure S2: EDS Graph, Figure S3: PL spectra, Figure S4: Tauc Plot representation, Figure S5: Lifetime and ET efficiency of CsPbCl3/Yb3+/xNd3+ PeNCs, Figure S6: UPS spectra cut-off region (b) Band-edge region (c). Luminance Intensity of PeNCs. Table S1: Bi-exponential fitting results.
