# Supplementary material for: Synergistic Enhancement of Near-Infrared Emission in CsPbCl3 Host via Co-Doping with Yb3+ and Nd3+ for Perovskite Light Emitting Diodes
Source: Nanomaterials (Basel). 2023 Oct 4;13(19):2703. doi: 10.3390/nano13192703 (PMC10574356; doi:10.3390/nano13192703)
Supplement: Supplementary file 1 [file nanomaterials-13-02703-s001.zip › nanomaterials-2602866-supplementary.pdf]

Synergistic Enhancement of Near-Infrared Emission in $CsPbCl_3$ Host via Co-Doping with Yb^{3+} and Nd^{3+} for Perovskite Light Emitting Diodes

Muhammad Amin Padhiar [1,2,3], Shaolin Zhang [1,2,3*], Minqiang Wang [4], Noor Zamin Khan [1,2], Shoaib Iqbal [4], Yongqiang Ji [4,5], Nisar Muhammad [6], Syed Ali K han [7] and Shusheng Pan [1,2,3*]

[1]School of Physics and Materials Science, Guangzhou University, Guangzhou 510006, China;

amin.padhiar@gzhu.edu.cn (M.A.P.); noorzamin@mail.ustc.edu.cn (N.Z.K.)

[2]Research Center for Advanced Information Materials (CAIM), Huangpu Research and Graduate School of Guangzhou University, Guangzhou 510555, China

[3]Key Lab of Si-based Information Materials & Devices and Integrated Circuits Design, Department of Educa-tion of Guangdong Province

[4]Electronic Materials Research Laboratory, Key Laboratory of the Ministry of Education International Center for Dielectric Research Xi'an Jiaotong University, Xi'an 710049, China; mqwang@xjtu.edu.cn (M.W.).

shoaibiqbalxjtu@outlook.com (S.I.); yongqiangji@pku.edu.cn (Y.J.)

[5]State Key Laboratory for Artificial Microstructure and Mesoscopic Physics, School of Physics,

Peking University, Beijing 100871, China

[6]Hefei National Research Center for Physical Sciences at the Microscale and Department of Physics,

University of Science and Technology of China, Hefei 230026, China; lai-ba@mail.ustc.edu.cn

[7]Department of Chemistry and Chemical Engineering, Rutgers, The State University of New Jersey,

08854 , USA; sayedali.khan@rutgers.edu

*Correspondence: slzhang@gzhu.edu.cn (S.Z.); sspan@gzhu.edu.cn (S.P.)

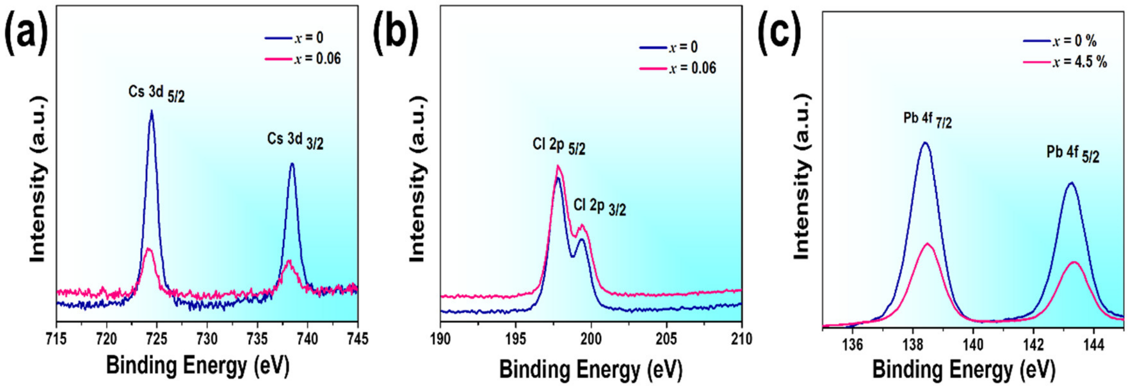

Figure. S1 High resolution XPS graph of $CsPbCl_3/Yb^{3+}/xNd^{3+}$ PeNCs (x = 0, 0.06) with elements Cs, Cl and Pb respectively.

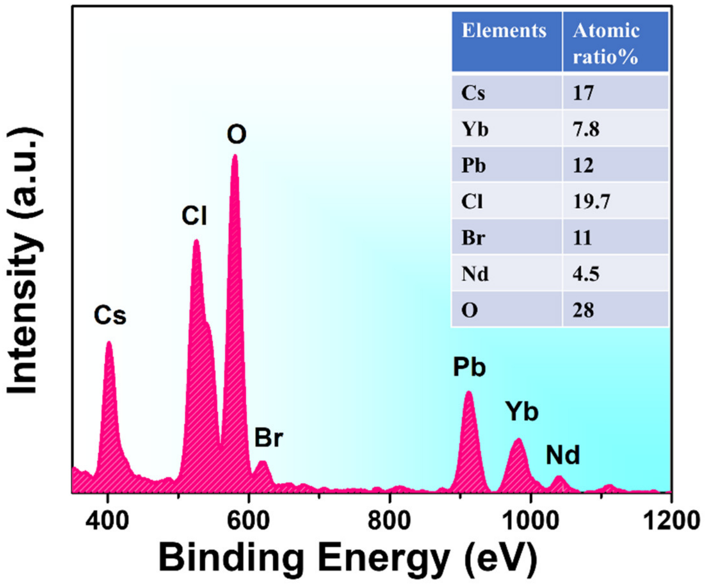

Elements	Atomic ratio%
Cs	17
Yb	7.8
Pb	12
Cl	19.7
Br	11
Nd	4.5
O	28

Figure. S2 EDS graph with atomic percentage of each element present in $CsPbCl_3/Yb^{3+}/xNd^{3+}$ PeNCs (x = 0.06).

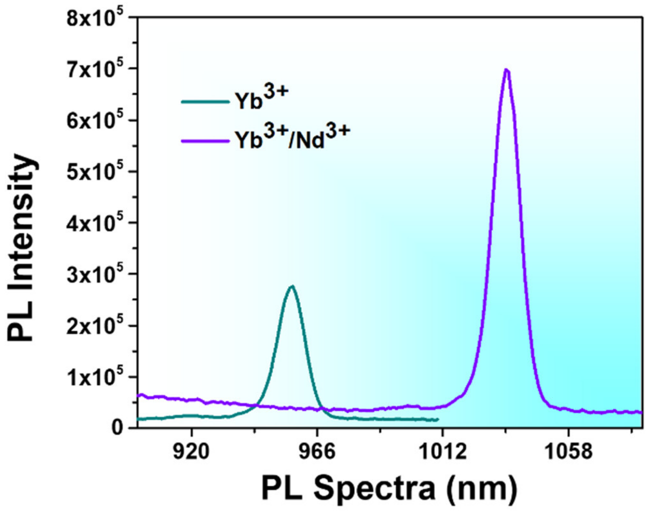

Figure S3. PL spectra of Single doped and Co-doped PeNCs (x=0, 0.06)

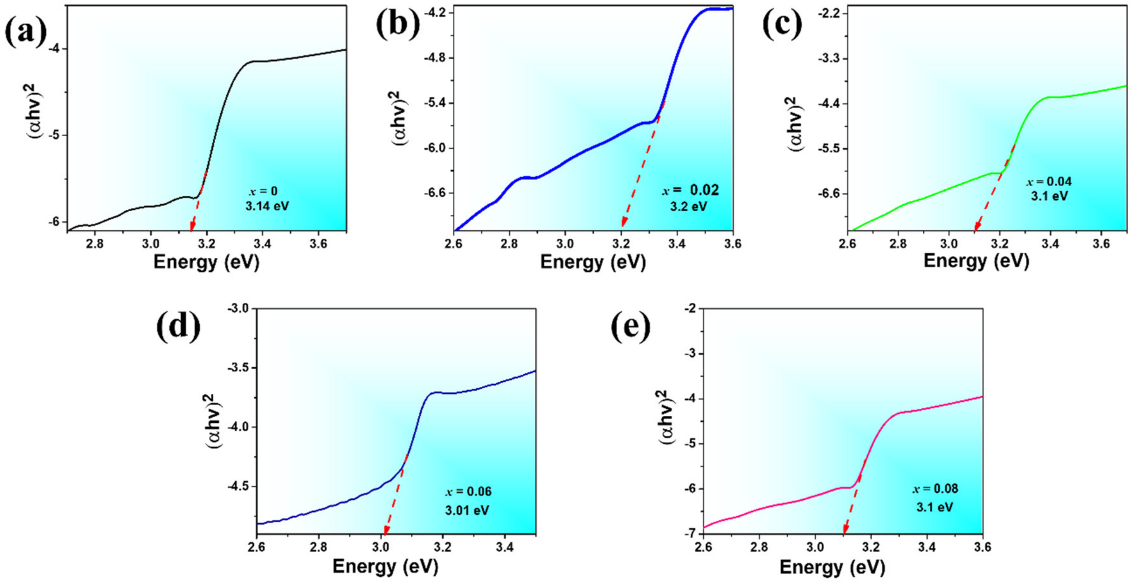

Figure. S4 Tauc Plot representation showing bandgap of $CsPbCl_3/Yb^{3+}/xNd^{3+}$ PeNCs (x=0, 0.02, 0.04, 0.06, 0.08) respectively.

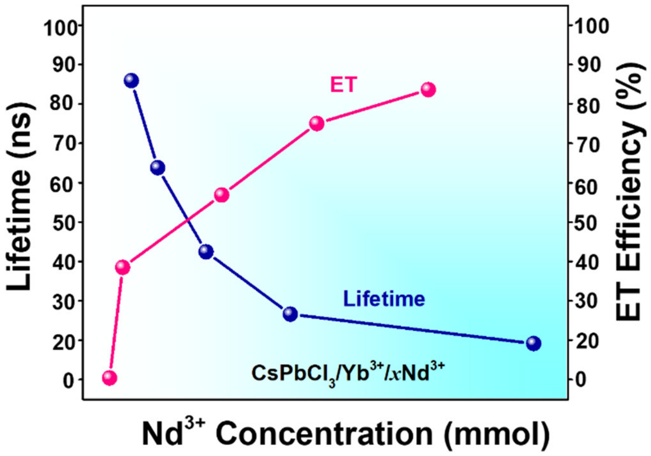

Figure. S5 Lifetime and ET efficiency of $CsPbCl_3/Yb^{3+}/xNd^{3+}$ PeNCs (x=0, 0.02, 0.04, 0.06, 0.08)

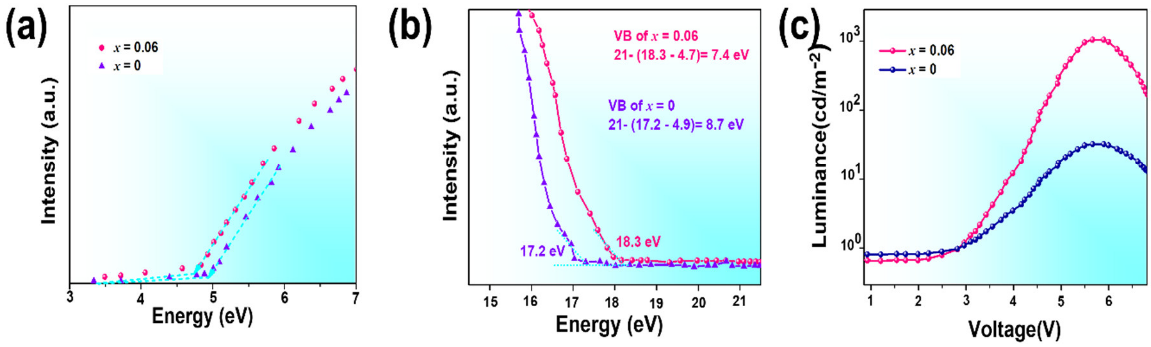

Figure. S6 UPS spectra cut-off region(b) Band-edge region. (c) Luminance Intensity of PeNCs (x=0, 006)

Table S1 Bi-exponential fitting results of the time resolved PL spectra of $CsPbCl_3/Yb^{3+}/xNd^{3+}$ PeNCs treated by different contents of Nd^{3+} (x=0, 0.02, 0.04, 0.06 and 0.08) respectively

x = 0	x = 0.02	x = 0.4	x = 0.06	x = 0.08
τ_1=15.01 ns	τ_1=13.46 ns	τ_1=12.24 ns	τ_1=11.05 ns	τ_1=10.66 ns
B_1%=70.53	B_1%=88.64	B_1%=92.48	B_1%=95.44	B_1%=96.59
τ_2=121.2 ns	τ_2=86.2 ns	τ_2=79.25 ns	τ_2=76.23 ns	τ_2=74.64 ns
B_2%=30.45	B_2%=18.9	B_2 %=15.46	B_2%=14.69	B_2%=11.82
τ_{avg}=47.48 ns	τ_{avg}=28.22 ns	τ_{avg}= 23.57ns	τ_{avg}=21.73 ns	τ_{avg}= 19.0 ns
